# QSAR and Free–Wilson Analysis of Bioisosteric Derivatives of Dimebon to Optimize the Search for Potential Inhibitors of Mitochondrial Permeability Transition

**DOI:** 10.3390/molecules31203775

**Published:** 2026-10-21

**Authors:** Ivan M. Veselov, Denis V. Zverev, Dmitry A. Shulga, Alexey Yu. Aksinenko, Elena R. Milaeva, Vladimir P. Fisenko, Elena F. Shevtsova, Sergey O. Bachurin

**Affiliations:** 1 Institute of Physiologically Active Compounds at Federal Research Center of Problems of Chemical Physics and Medicinal Chemistry, Russian Academy of Sciences (IPAC RAS), Severny Proezd 1, Chernogolovka 142432, Russia; jowent@mail.ru (I.M.V.); alaks@ipac.ac.ru (A.Y.A.); shevtsova@ipac.ac.ru (E.F.S.); 2 Department of Medicinal Chemistry and Fine Organic Synthesis, Faculty of Chemistry, Lomonosov Moscow State University, Leninskie Gory, 1, Building 3, Moscow 119991, Russia; denzverev@qsar.chem.msu.ru (D.V.Z.); shulga@qsar.chem.msu.ru (D.A.S.); milaeva@med.chem.msu.ru (E.R.M.); 3 Department of Pharmacology, I.M. Sechenov First Moscow State Medical University (Sechenov University), Ministry of Health of Russian Federation, 8 Trubetskaya St., Moscow 119991, Russia; vpfisenko@mail.ru

**Keywords:** mitochondrial permeability transition, QSAR, Free–Wilson, structure–activity relationships

## Abstract

Mitochondrial permeability transition pore (MPTp) opening is a critical event in neurodegeneration, representing a point of no return in cell death cascades. Despite the neuroprotective potential of MPTp inhibitors, systematic structure–activity relationship (SAR) studies for this target remain scarce. Here we present the first standardized dataset of 57 γ-carboline derivatives evaluated for their ability to inhibit MPTp under uniform experimental conditions. Using complementary Free–Wilson and 2D-QSAR analyses, we expertly propose rational structure–activity relationships (SAR) for this scaffold. Based on the Free–Wilson analysis, we assume that increasing lipophilicity and steric bulk at all four substitution positions to a certain limit positively correlates with inhibitory activity. The R3 linker plays a critical role: the presence of positively ionizable amine groups and potentially hydrogen bond donors enhances activity, whereas non-ionizable amide linkers may be detrimental. The QSAR model based on physicochemical descriptors of substituents corroborates these findings, with positive contributions from cLogP and HBD descriptors. This integrated computational–experimental approach provides a predictive framework for the rational design of novel neuroprotective agents targeting MPTp, with potential implications for the treatment of neurodegenerative diseases.

## 1. Introduction

The importance of mitochondria as a key factor in the pathogenesis of neurodegenerative diseases (NDD) and, accordingly, as a target for the search for potential neuroprotectors, is currently quite multifaceted but beyond doubt [1,2,3,4]. In this regard, a special place is occupied by the process of opening of mitochondrial permeability transition pores (MPTp), which is a key process and a point of no return in cell death cascades [5,6]. Disturbances in mitochondrial functions and metabolic activity of the brain in neurodegenerative diseases are detected long before the onset of symptoms [7,8], but what is more important is that the death of neurons during the development of neurodegenerative processes is largely associated with an increase in the sensitivity of brain mitochondria to the induction of the opening of the MPTp [9,10]. This is a characteristic feature not only of the aging brain, but also, to a much greater extent, of the result of the action of various factors in the pathogenesis of NDD, including neurotoxins and specific proteins of proteinopathies [11,12].

One of the first drugs for which it was shown that the neuroprotective effect is largely associated with the modulation (inhibition) of the opening of the MPTp is dimebon (latrepirdine) [13,14]. The neuroprotective activity of dimebon was first demonstrated under conditions of β-amyloid toxicity in a primary culture of rat cerebral cortex neurons [15], but dimebon also effectively suppresses both β-amyloid- and calcium-induced opening of the MPTp of isolated mitochondria from rat brain and liver [14].

Over the past ten years, various derivatives of dimebon have been synthesized and studied, including fluorine-containing derivatives, conjugates of γ-carbolines with peptide residues, aminoadamantanes and phenothiazines [16,17,18]. One of the key characteristic activities of these compounds is inhibition of the MPTp-opening process. In each of the described groups of γ-carboline derivatives, the most active compounds were isolated, and the observed effects of various molecular modifications were described. However, a systematic structure–activity analysis has not been conducted. This analysis makes it possible to formulate well-founded hypotheses regarding the nature of ligand interactions with potential targets and to propose potential strategies for optimizing potential MPTp inhibitor candidates.

Retrospective QSAR analysis [19,20] and Free–Wilson analysis [21] provide powerful computational tools for deconstructing how specific physicochemical and structural patterns may presumably influence the biological activity of compounds. While QSAR methods leverage global molecular properties (e.g., lipophilicity, polarity, steric bulk) to build predictive models, the Free–Wilson approach offers a more intuitive, fragment-based perspective by assigning additive activity contributions to individual substituents. The complementary application of these two methods is particularly valuable in cases where the molecular target is unknown or forms a difficult-to-isolate multiprotein complex, such as the MPTp. This approach enables one to propose the strategy of rational, data-driven lead optimization without requiring detailed knowledge of the receptor’s three-dimensional structure at early stages of the investigation. In the present study, we applied both methodologies to a series of γ-carboline derivatives to infer key structural determinants of MPTp inhibitory activity. Moreover, the integration of fragment-based contributions (Free–Wilson) with property-based predictions (QSAR) allows us to suppose both “hot spots” for substitution and global physicochemical thresholds for activity, thereby providing a comprehensive roadmap for further medicinal chemistry optimization.

Therefore, the task was set for the existing series of γ-carboline derivatives (fluorine-containing derivatives of dimebon and conjugates of γ-carbolines with aminoadamantanes) to conduct screening studies of their effect on the induction of MPT under optimized conditions and, based on the data obtained, to perform a retrospective QSAR analysis and Free–Wilson analysis to propose MPTp inhibitory activity patterns.

## 2. Results and Discussion

### 2.1. Screening Assay Details

Previously, the effects of representatives of different classes of γ-carboline dimebon derivatives were assessed under various conditions, using isolated liver and brain mitochondria, in the presence of different mitochondrial respiratory chain substrates, etc. Such heterogeneity does not allow for a sufficiently accurate comparison of their activities as γ-carboline inhibitors, especially given the previously demonstrated dependence of γ-carboline dimebon MPTp inhibition activity on mitochondrial respiratory chain activity and the absence of inhibition under conditions of mitochondrial deenergization [14].

Therefore, the first objective of this study was to conduct screening studies to assess the effect of fluorine-containing derivatives of dimebon and conjugates of γ-carbolines with aminoadamantanes (57 compounds, Supplement S1) on the opening of MPTp. Experiments were carried out on isolated rat liver mitochondria, since, unlike brain mitochondria, they represent a more homogeneous population with a pronounced “swelling” reaction in response to the addition of calcium ions to the medium, which is a marker of changes in the shape of mitochondria as a result of the process of opening the MPTp. To energize mitochondria, both NADH- and FADH-dependent substrates were present in the medium: glutamate with malate and succinate.

**Figure 1:**
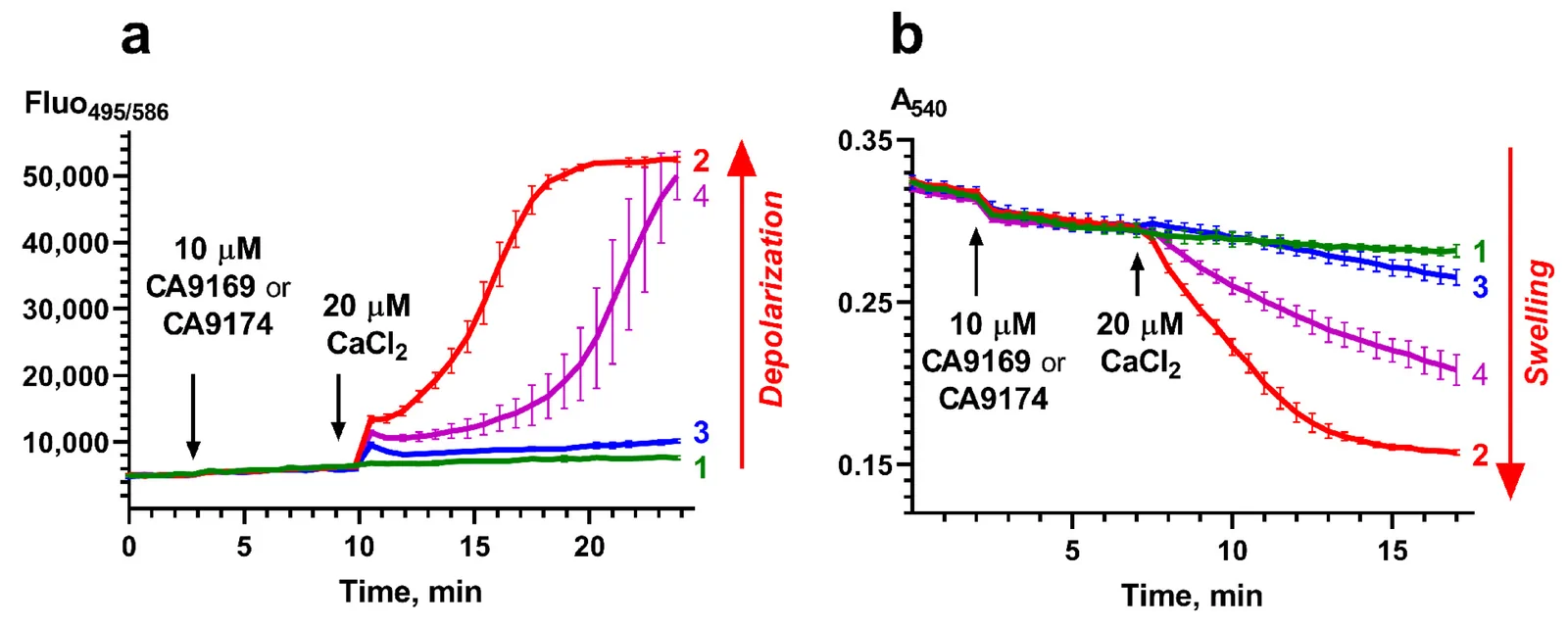
Representative graphs of the determination of (**a**) mitochondrial potential and (**b**) mitochondrial swelling. Additions for 1—solvent without CaCl_2_; 2—solvent and 20 µM CaCl_2_; 3—10 µM CA9169 and 20 µM CaCl_2_; 4—compound 10, µM CA9174 and 20 µM CaCl_2_.

The first step was to assess the effect of the compounds on mitochondrial potential, determined using the voltage-dependent fluorescent marker safranin O (Figure 1a). This makes it possible to identify compounds that can depolarize mitochondria as a result of inhibition of mitochondrial respiratory chain complexes or uncoupling of the process of oxidative phosphorylation. Next, the kinetics of absorption (λ540) of the mitochondrial suspension were assessed to identify compounds that could cause changes in shape of the mitochondria, in particular by inducing or preventing the mitochondrial swelling caused by calcium-induced MPTp opening (Figure 1b).

Among the studied compounds, no compounds were found that caused “swelling” or depolarization of mitochondria without the addition of calcium. Based on the results of earlier work, the optimal concentration of compounds was chosen—10 μM. At this concentration, the rates of mitochondrial swelling in the presence of all the compounds studied were in the range between the values of the blank group (without calcium addition) and the control group (with calcium addition). The degree of inhibition of the mitochondrial swelling rate is presented in the table (Supplementary Materials S1).

We would like to note that the set of 57 compounds containing the gamma-carboline pharmacophore and exhibiting MPTp inhibitory activity, which we used in this study, is the most extensive to date. Furthermore, data on the effects of all these compounds on calcium-induced swelling were obtained using a sufficiently homogeneous preparation of isolated rat liver mitochondria under standardized conditions of mitochondrial energization, employing substrates for respiratory chain complexes I and II. Both expert and statistical analyses can be used further to establish the structure–activity relationships (SAR) to rationalize the observed activity and to provide hints for further development.

Having acquired the mitochondrial swelling under standardized experimental conditions, we conducted a Free–Wilson analysis to assess the contributions of molecular fragments to activity, as well as a QSAR analysis using molecular descriptors. The results allowed for expert analysis of a deeper understanding of the observed trends in structure–activity relationships within these complex mitochondrial systems.

### 2.2. Free–Wilson Analysis

We performed a Free–Wilson (FW) analysis on the synthesized molecule series to investigate whether the SAR is additive or not. This linear regression method enables determination of the substituents’ contributions to the overall compound activity, with the derived coefficients serving to predict the activity of new chemical entities.

**Scheme 1:**
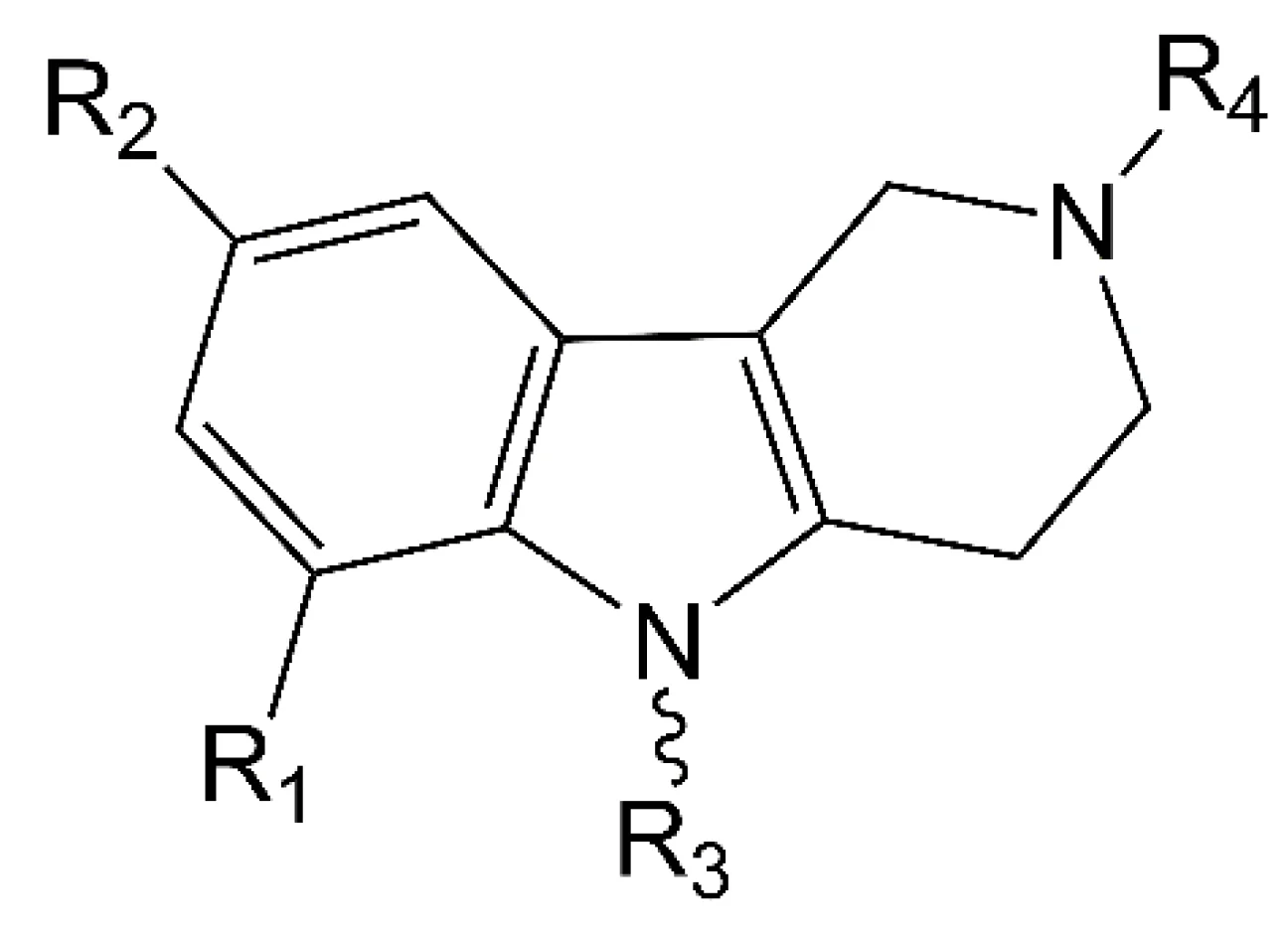
The core 1,2,3,4-tetrahydro-γ-carboline scaffold used for the study.

The molecules were divided into fragments based on the common 1,2,3,4-tetrahydro-γ-carboline core; all non-hydrogen substituents were at positions 3,6,8,9 and were designated as R4, R2, R1 and R3 groups, respectively (Scheme 1). The FW model was generated by fitting a generalized linear model coefficients to the observed mitochondrial swelling activity data, employing the substituents (Ri) as the descriptor variables. The calculated core activity was used as a bias term.

**Figure 2:**
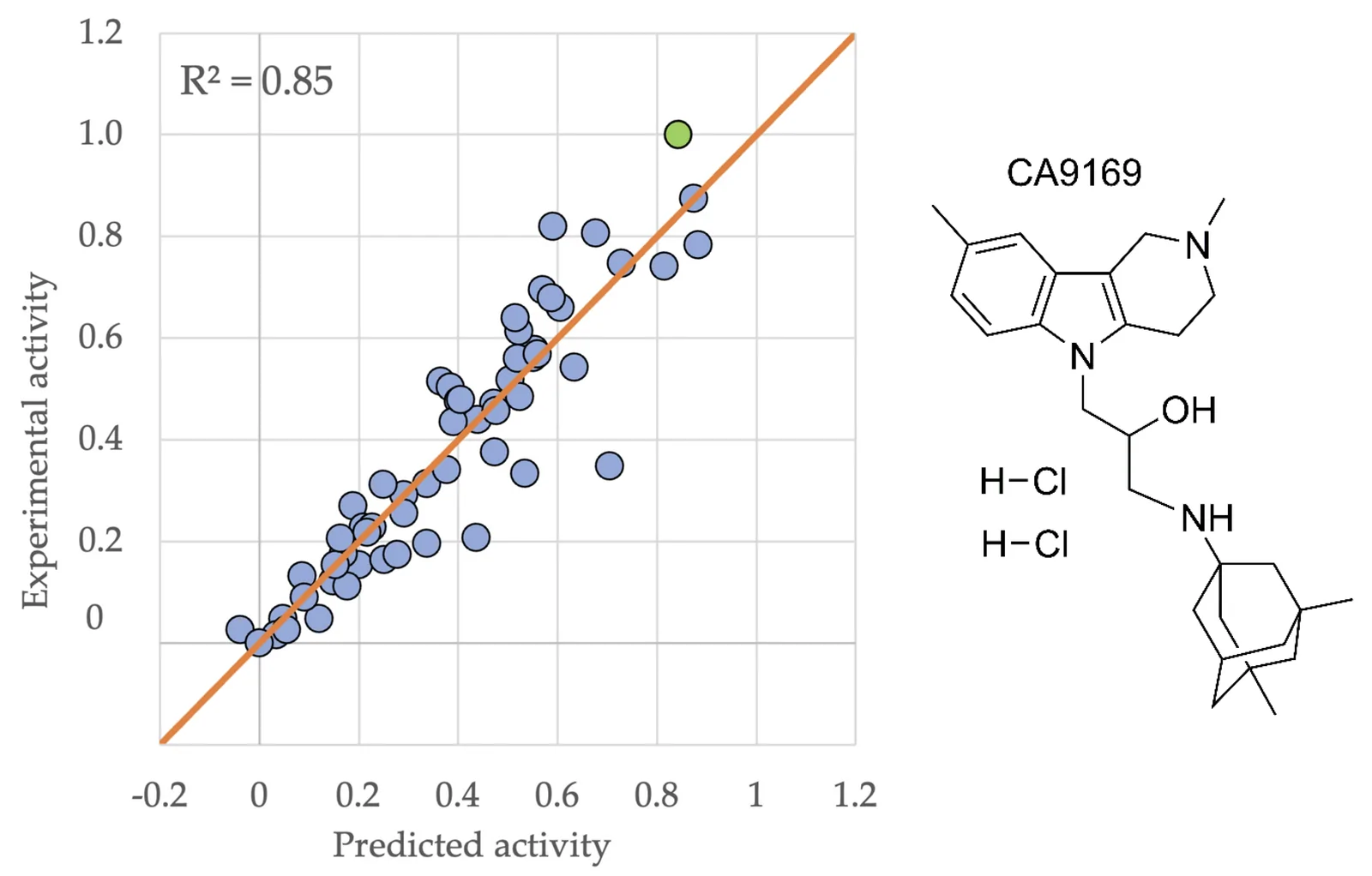
Plots of measured vs. calculated mitochondrial swelling activity values (normalized to the CA9169 value (green dot; structure shown on the right) from the Free–Wilson analysis.

Mitochondrial swelling percentages for the series of synthesized compounds were normalized to the corresponding value obtained for the most active compound CA9169 (Figure 2).

The calculated coefficients for substituents R1 and R4 are presented in Table 1 relative to the coefficient of the hydrogen substituent in the R1 group and the methyl substituent in the R4 group as the reference values. For these substituents, a general trend toward increasing activity with increasing lipophilicity and substituent size is observed. Moreover, at position R4, the largest positive coefficient corresponds to the longer propyl substituent, rather than the bulkier isopropyl substituent. This may indicate a positive effect of the aliphatic substituent length, provided the optimal steric volume is maintained. However, the SAR data are insufficient for an unambiguous analysis of the contributions of groups at these positions, especially for position R1. Therefore, further study of other series of 3,8-substituted 1,2,3,4-tetrahydro-γ-carbolines is required to establish a more comprehensive SAR.

The calculated coefficients for the R2 substituents are shown in Table 2 relative to the hydrogen. This group also shows a tendency for activity to increase with increasing substituent size, which is most clearly evident in the halogen and alkyl substituent series. However, a U-shaped size–activity relationship may be observed at this position, as evidenced by the low coefficient for the bulkier trifluoromethyl substituent; this suggests a requirement for complementarity with the binding pocket’s volume. Bromine and isopropyl demonstrate the largest and comparable contributions at different steric volumes, which may indicate not only a positive effect of substituent bulk, but also the presence of additional interactions. The observed activity trend F < Cl < Br at the R2 position is consistent with increasing lipophilicity and polarizability, and may also be rationalized by potential “halogen bonding” interactions, as suggested for related systems [22,23,24]. However, further structural studies would be required to confirm this hypothesis. The negative coefficient of the methoxy substituent, comparable in size to ethyl, indicates the decisive influence of lipophilicity, as well as the possible undesirable presence of a hydrogen bond acceptor at this position. As for positions R1 and R4, additional expansion of substituents looks a promising strategy to establish a clearer SAR and the boundaries to group expansion.

The calculated coefficients for the R3 substituents relative to the hydrogen are shown in Table 3. This position is the most extensively and diversely substituted in the series of compounds analyzed. The pyridine-containing substituents exhibit moderately positive coefficients, suggesting the importance of the lipophilicity and the bulk of the fragment at the end of the linker. This assumption is also supported by the highest coefficient in this subgroup for the substituent containing the bulkier 2-trifluoromethylpyridine fragment. A moderately positive contribution is demonstrated by the vinyl substituent, which is relatively small but quite lipophilic. In contrast, the substituent containing the ethyltriazole fragment has a negative coefficient, consistent with its insufficient bulk and lipophilicity.

A series of substituents containing adamantyl fragments exhibits a significant positive contribution to activity. Moreover, substituents containing the bulkiest dimethyladamantyl moiety exhibit larger coefficient values compared to similar substituents without methyl groups. This is also consistent with the assumption of the importance of the linker-terminal fragment size. Pairwise analysis of coefficients of substituents with identical functionality but different linker lengths allows us to determine the optimal value of four linear atoms. The presence of a positively ionizable group (e.g., a secondary amine) in the linker also indicates a significant increase in activity. Conversely, substituents with non-ionizable amide linkers have coefficients that are two or more times lower than similar substituents. The low activity of compounds with an amide linker also presumably indicates a negative impact of a hydrogen bond acceptor at position 2 relative to the core. On the contrary, the presence of a hydrogen bond donor group at position 2 of the linker may make a positive contribution to activity. Despite the observation that the possible importance of the positively ionizable group follows only indirectly from the Free–Wilson analysis, it is consistent with the medicinal chemist expert view. First, the presence of such positive groups helps to balance the bulky and lipophilic nature of the core compounds and its substituents. Second, such a pattern is well-known for agonists and antagonists of GPCRs related to the central nervous system (CNS).

The abnormally high coefficient of the benzyl substituent is inconsistent with the general trends in the R3 group. This result may be either a statistical outlier or a possible indication of specific anchoring interactions with the benzene ring. For a more definitive analysis, a larger number of compounds containing a benzyl fragment in this position are needed.

### 2.3. QSAR Analysis

**Figure 3:**
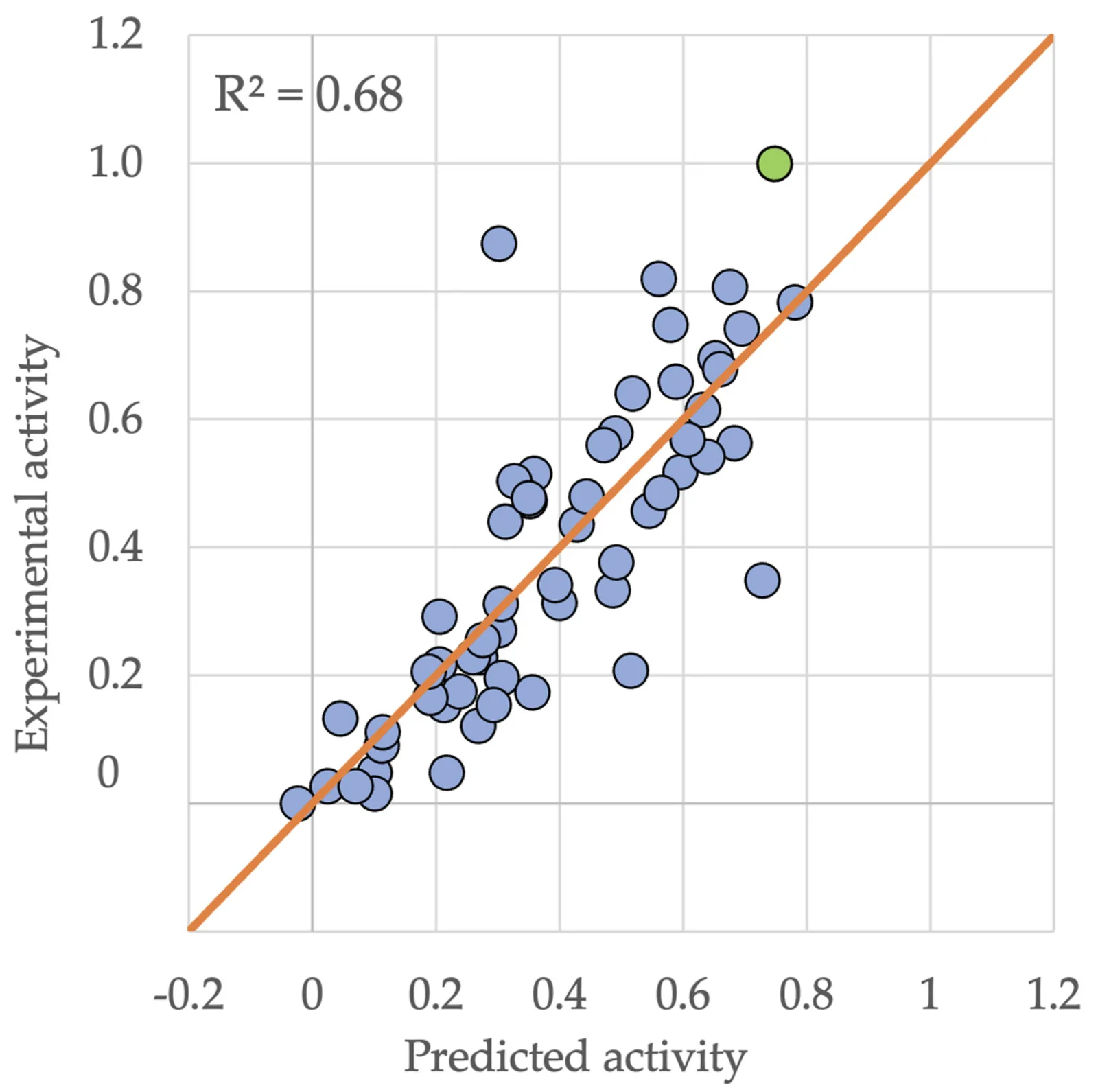
Plots of measured vs. calculated mitochondrial swelling activity values (normalized to the CA9169 value (green dot) from the 2D-QSAR descriptors model.

The 2D-QSAR investigation comprised five molecular descriptors: calculated logP (ClogP), heavy atom count (HAC), number of hydrogen bond donors (HBD), number of hydrogen bond acceptors (HBA), and topological polar surface area (TPSA), which were calculated separately for each substituent. Normalized descriptor values were concatenated into single vectors. A multivariate linear regression model was constructed for these vectors as variables (Figure 3). This method made it possible to determine the contribution of each descriptor to the overall activity for each substituent position. This approach allowed us to generalize the patterns of substituent contributions to activity with more structurally independent physicochemical parameters, making it possible to obtain more generalized SAR hypotheses. The calculated coefficients for the descriptors are shown in Table 4.

For the R1 and R4 groups, it is not possible to make a reliable prediction of the descriptor model due to the small number of compounds substituted at these positions. The R1 data correspond to a single compound, while the R4 data are inconsistent with the Free–Wilson analysis. The HBD, HBA, and TPSA descriptors have zero coefficients due to the absence of polar atoms at these positions. For the R2 group, a significant positive contribution of cLogP is observed, consistent with the results of FW analysis and the increasing activity contributions in the halogen substituent series. The negative HAC coefficient is due to the low contribution of trifluoromethyl to the activity compared to monoatomic halogen substituents, as well as the negative contribution of the methoxy fragment. The small positive TPSA coefficient corresponds to the positive contributions of the halogen series, but is offset by the negative value for the methoxy group.

For the most diverse series of R3 substituents, the QSAR descriptor model agrees well with the Free–Wilson analysis. The high positive cLogP coefficient and the small negative TPSA coefficient confirm the importance of the lipophilic substituent. The negative HAC coefficient may correspond to an abnormally high contribution of the benzyl substituent. The high positive HBD coefficient is consistent with the importance of having predominantly hydrogen bond donors in the linker, such as a positively ionizable amino group or hydroxyl group in the series of active adamantyl-containing substituents. However, this descriptor is partially correlated with HBA, which explains the moderate positive coefficient of the latter.

## 3. Materials and Methods

### 3.1. Synthesis

References to the synthesis and properties of compounds described previously and a description of the synthesis and properties of new compounds are provided in the Supplementary Materials.

### 3.2. Free–Wilson Analysis Procedure

The R-group decomposition of the compound library was performed using the rdRGroupDecomposition module implemented in the RDKit software suite (version 2026.03.3) [25]. Ridge regression was used to establish the linear regression dependency of the presence of each substituent in each defined position in Free–Wilson analysis. The experimental activities were normalized to the range of [0, 1] using the minimum and maximum activity in the set, respectively. The constructed model is used exclusively to yield quantitative parameters that facilitate expert analysis and interpretation of SAR and is not intended to predict new, potentially active compounds. Due to this, and the small amount of data, it was intentionally decided not to use validation methods. Therefore, no division into training and test sets was performed.

### 3.3. QSAR Descriptors Model

The 2D-QSAR investigation comprised five molecular descriptors: calculated logP (ClogP), heavy atom count (HAC), number of hydrogen bond donors (HBD), number of hydrogen bond acceptors (HBA), and topological polar surface area (TPSA), which were calculated separately for each substituent. Normalized descriptor values were concatenated into single vectors. A multivariate linear regression model was constructed for these vectors as variables. The descriptors were calculated using the RDKit software suite (version 2026.03.3) [25] with the following correspondence to RDKit descriptor functions: cLogP—logP(), HAC—HeavyAtomCount(), HBD—NumHDonors(), HBA—NumHAcceptors(), and TPSA—TPSA().

The values of the descriptors obtained on the dataset were normalized to z-scores (zero mean and unity standard deviation) using the StandardScaler class from the scikit-learn library [26] in order to estimate the relative importance of descriptors.

Based on the reasons stated in Section 3.2, no division into train and test sets or subsequent model validation was performed.

### 3.4. Experimental Biological Assays

#### 3.4.1. Isolation of Rat Liver Mitochondria

Outbred male rats weighing 200–220 g (Stolbovaya Breeding Center, Chekhov, Russia) were used in the experiments. The animals were maintained in a standard vivarium with a 12 h light cycle and free access to food and water. The standard procedures for rat euthanasia with carbon dioxide used in this study were approved by the Bioethics Committee of the IPAC RAS (Protocol No. 72 dated 24 April 2023).

Rats were euthanized in a desiccator in a carbon dioxide atmosphere. After rapid decapitation using a guillotine (OOO NPK Open Science, Moscow, Russia), the abdominal cavity was opened and the livers were removed into ice-cold (1:1) isolation buffer (260 mM sucrose, 10 mM HEPES, 1 mM EGTA, pH 7.4). The liver was then minced with scissors, washed, and homogenized in buffer (1:5) in a Potter homogenizer (Sartorius AG, Göttingen, Germany) at 350 rpm (7 full up-and-down strokes). The mitochondrial fraction was obtained by differential centrifugation: the homogenate was initially centrifuged in a Rotina centrifuge (Hettich, Kirchlengern, Germany) at 1500 g, 4 °C for 13 min to sediment the heavy fraction of nuclei and intact cells, after which the collected supernatant was centrifuged at 10,570 g, 4 °C for 13 min to sediment the mitochondria. The supernatant was removed and the obtained fraction was washed in 25 mL of ice-cold wash buffer (260 mM sucrose, 10 mM HEPES, 100 μM EGTA, pH = 7.4), after which the mitochondrial pellet was diluted in 1 mL of wash buffer and stored for 2–4 h at 4 °C. The protein concentration in the resulting mitochondrial suspension was determined by the standard biuret method.

#### 3.4.2. Measurement of Mitochondrial Potential

To exclude compounds that depolarize mitochondrial membranes and thereby inhibit MPTp opening, their effect on mitochondrial potential was assessed. Measurements were performed using the cationic dye safranin O, which penetrates mitochondria energized by the addition of respiratory chain substrates, leading to quenching of its fluorescence. The measurement medium contained 260 mM sucrose, 1 mM KH_2_PO_4_, substrates (10 mM glutamate, 2 mM malate, and 5 mM succinate), and 10 μM safranin O. A mitochondrial suspension (final protein concentration: 0.5 mg/mL) was added to the medium. The resulting working suspension was pipetted into the wells of an opaque 96-well plate, and fluorescence was measured using an EnVision plate reader (PerkinElmer Wallac Oy, Helsinki, Finland) with excitation at 495 nm and detection at 586 nm. During the measurement, the test substances were added, followed by CaCl2 at the same concentration as in the swelling assay (Figure 1a). The resulting fluorescence values were normalized to the initial point (0%) and the fluorescence value after reaching a plateau after the addition of calcium chloride (100% depolarization) and defined as the degree of depolarization.

#### 3.4.3. Measuring the Rate of Mitochondrial Permeability Pore Opening as Mitochondrial Swelling

The MPTp opening process was assessed spectrophotometrically by measuring the decrease in absorbance of the mitochondrial suspension. The measurement medium contained 260 mM sucrose, 1 mM KH_2_PO_4_, and substrates (10 mM glutamate, 2 mM malate, and 5 mM succinate), at pH 7.2. The mitochondrial suspension was added to the medium (final protein concentration: 0.5 mg/mL). The resulting working suspension was pipetted into the wells of a transparent 96-well plate, and the absorbance of the samples was measured at 540 nm using an EnVision plate reader (PerkinElmer Wallac Oy, Helsinki, Finland). During the measurement, the test substances were added at a concentration of 10 μM to assess their direct effect on pore opening. CaCl_2_ was then added to the samples at a concentration of 20–30 μM, causing a decrease in light absorbance of approximately 0.02 units per minute (Figure 1b). The results were processed to obtain the percent of inhibition of mitochondrial swelling rate in the presence of the test compounds, normalized to the swelling rates in the control group with and the blank group without addition of calcium chloride, which were taken as 0% and 100% of MPTp inhibition, respectively. Data are presented as MEAN ± SD; each value was obtained in triplicate on three mitochondrial preparations.

## 4. Conclusions

This study presents the first standardized dataset of 57 γ-carboline derivatives evaluated for their ability to inhibit mitochondrial permeability transition pore (MPTp) opening under uniform experimental conditions. The consistency of the biological data has enabled a retrospective analysis using complementary Free–Wilson and 2D-QSAR approaches, yielding rational structure–activity relationship (SAR) hypotheses for this scaffold.

The complementary nature of the two approaches is particularly valuable: Free–Wilson analysis allows one to predict the influence of each unique substituent on the structure’s activity, and the QSAR model complements these data with underlying easily interpretable physicochemical drivers (lipophilicity, hydrogen bonding capacity), providing a more generalized framework for future rational compound design.

The Free–Wilson analysis allows us to assume that the rationality of increasing lipophilicity and steric bulk to a certain limit at all four substitution positions (R1–R4) positively correlates with inhibitory activity. This observation suggests that the possible ligand-binding environment within the MPTp complex (e.g., the hydrophobic channels of cyclophilin D or F_O_/F_1_-ATP synthase) is predominantly hydrophobic, where enhanced van der Waals contacts are favored. Notably, the increased molecular size also offers a strategic advantage in reducing the promiscuity inherent to small, planar aromatic cores, potentially improving target specificity. However, a more detailed investigation into the dependence of activity on substituent size and the presence of potential additional interactions at positions R1, R2, and R4 requires further study of more diverse series of substituted 1,2,3,4-tetrahydro-γ-carbolines.

A particularly valuable finding in this analysis is the critical role of the R3 linker. The presence of positively ionizable amine groups and probably hydrogen bond donors (such as hydroxyl group) in the R3 linker is associated with increased activity, whereas the non-ionizable amide linker may be detrimental. This trend is quantitatively captured by the QSAR model with an HBD coefficient equal to 0.257. The high cLogP coefficient correlates with positive activity enhanced upon introduction of bulky and hydrophobic groups as the terminal moiety at R3. The presence of a positively ionizable amine group also fits well to the hypothesis of F_O_/F_1_-ATP synthase being an essential molecular target for MPTp formation inhibition. The role of linker length in activity is also shown.

Although the current dataset is insufficient to define the optimal substituent size limits at each position, the established SAR trends confidently provide ligand interaction patterns with potential targets. Based on these rules, we propose that the activity of MPTp inhibitors is positively influenced by (i) extended hydrophobic substituents at R2 and R3, (ii) the presence of positively ionizable amine functionality in the R3 linker to provide hydrogen bond donor capacity (with possible positive influence of extra hydrogen bond donors, such as hydroxyl), and (iii) systematically varied terminal groups to probe the steric and lipophilic tolerance of the binding pocket.

In summary, the integrated approach with strictly standardized experiments and relevant computational analysis described herein proposes rational structure–activity relationships of existing γ-carboline MPTp inhibitors and provides a predictive framework for the rational design of novel neuroprotective agents. While the targeted design and synthesis of new γ-carbolines based on our proposed SAR hypotheses are out of scope of this study, the derived structural guidelines can facilitate the focused synthesis of optimized compounds, ultimately accelerating the development of effective therapies for neurodegenerative diseases. Verification of the proposed assumptions is a direction for planning our further research.

## Figures and Tables

**Table 1:** Calculated activity coefficients for the R1 and R4 substituents relative to hydrogen and methyl, respectively.

Substituent	R1 Activity Coefficient	R4 Activity Coefficient
F	0.216	-
Et	-	0.020
Pr	-	0.142
iPr	-	0.068

**Table 2:** Calculated activity coefficients for the R2 substituents relative to hydrogen.

R2 Substituent	Activity Coefficient
F	0.086
Cl	0.154
Br	0.437
Me	0.114
Et	0.173
iPr	0.405
CF_3_	0.055
MeO	−0.074

**Table 3:** Calculated activity coefficients for the R3 substituents relative to hydrogen.

R3 Substituent	Activity Coefficient
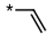	0.153
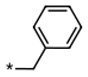	0.827
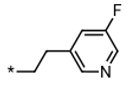	0.101
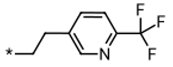	0.317
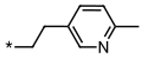	0.106
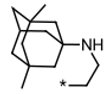	0.502
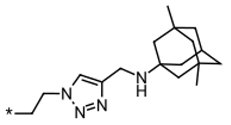	0.467
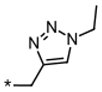	−0.019
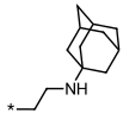	0.457
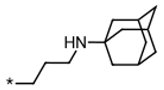	0.587
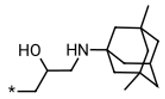	0.796
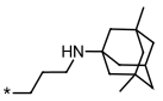	0.658
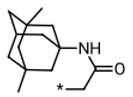	0.358
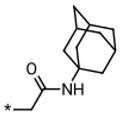	0.230
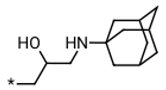	0.541

The asterisk (*) denotes the point of attachment to the core structure.

**Table 4:** Calculated activity coefficients for the molecular descriptors for each substituent position.

Descriptor	R1	R2	R3	R4
cLogP	0.086	0.317	0.146	−0.011
HAC	0.124	−0.102	−0.013	−0.027
HBD	0	0	0.257	0
HBA	0	0	0.113	0
TPSA	0	0.001	−0.009	0

## Data Availability

The original contributions presented in this study are included in the article/Supplementary Materials. Further inquiries can be directed to the corresponding author.

## Supplementary Materials

The following supporting information can be downloaded at https://www.mdpi.com/article/10.3390/molecules31203775/s1. The authors have cited additional references within the Supporting Information [27,28,29,30,31,32,33].

## Abbreviations

The following abbreviations are used in this manuscript:

MPTp	Mitochondrial permeability transition pore
SAR	Structure–activity relationship
NDD	Neurodegenerative diseases
FW	Free–Wilson
CNS	Central nervous system
HAC	Heavy atom count
HBD	Hydrogen bond donors
HBA	Number of hydrogen bond acceptors
TPSA	Topological polar surface area
