# Supplementary material for: QSAR and Free–Wilson Analysis of Bioisosteric Derivatives of Dimebon to Optimize the Search for Potential Inhibitors of Mitochondrial Permeability Transition
Source: Molecules. 2026 Oct 21;31(20):3775. doi: 10.3390/molecules31203775 (PMC13707804; doi:10.3390/molecules31203775)
Supplement: Supplementary file 1 [file molecules-31-03775-s001.zip › molecules-4596216-supplementary.pdf]

Supplement (S1).

tetrahydro-carboline core

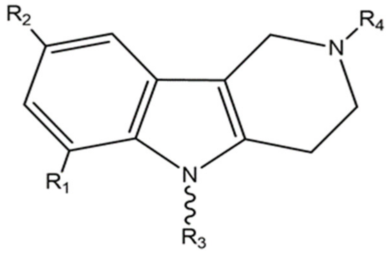

R3:

Pyridyl-3 (Py-3) Memantyl (Mem) Amantadyl (Ama)

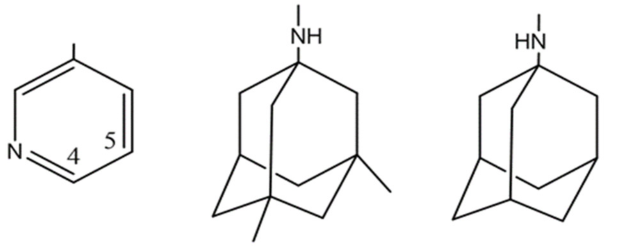

No.	Codes	R1	R2	R3	R4	Linker	Activity (inhibition of rate of swelling), %		Ref.
							MEAN	SD	
1	CA7677*	H	CH_3	-	CH_3	-	4,5	10,5	[31]
2	EC3037	H	CH_3	-	CH_3	$-CH=CH_2$	14,4	4,3	[27]
3	EC3039	H	H	-	CH_3	$-CH=CH_2$	12,4	8,4	[27]
4	CA8363	H	CH_3	phenyl	CH_3	$-CH_2-$	82,1	4,1	[32]
5	DF301	H	H	5-F-Py-3	CH_3	$-(CH_2)_2-$	1,5	6,6	[28]
6	DF302	H	CH_3	5-F-Py-3	CH_3	$-(CH_2)_2-$	11,4	6,4	[28]
7	DF303	H	F	5-F-Py-3	CH_3	$-(CH_2)_2-$	4,5	5,7	[28]
8	DF304	H	Cl	5-F-Py-3	CH_3	$-(CH_2)_2-$	25,4	6,0	[28]
9	DF305	H	Br	5-F-Py-3	CH_3	$-(CH_2)_2-$	44,3	2,6	[28]
10	DF306	H	CH_3O	5-F-Py-3	CH_3	$-(CH_2)_2-$	2,5	11,7	[28]
11	DF308	F	F	5-F-Py-3	CH_3	$-(CH_2)_2-$	29,3	6,2	[28]
12	DF309	H	CF_3	5-F-Py-3	CH_3	$-(CH_2)_2-$	8,4	11,9	[28]
13	DF311	H	H	5-F-Py-3	C_2H_5	$-(CH_2)_2-$	2,5	6,8	[28]
14	DF312	H	CH_3	5-F-Py-3	C_2H_5	$-(CH_2)_2-$	16,4	4,6	[28]

No.	Codes	R1	R2	R3	R4	Linker	Activity (inhibition of rate of swelling), %		Ref.
							MEAN	SD	
15	DF315	H	Cl	5-F-Py-3	C_2H_5	$-(CH_2)_2-$	21,4	5,7	[28]
16	DF316	H	C_2H_5	5-F-Py-3	C_2H_5	$-(CH_2)_2-$	21,4	5,8	[28]
17	DF318	H	CH_3	5-F-Py-3	iso-C_3H_7	$-(CH_2)_2-$	20,4	7,1	[28]
18	DF319	H	CH_3	5-F-Py-3	C_3H_7	$-(CH_2)_2-$	27,4	5,0	[28]
19	DF320	H	iso-C_3H_7	5-F-Py-3	CH_3	$-(CH_2)_2-$	41,3	9,0	[28]
20	DF401	H	H	4-CF_3-3-Pyr	CH_3	$-(CH_2)_2-$	15,4	8,2	[28]
21	DF402	H	CH_3	4-CF_3-3-Pyr	CH_3	$-(CH_2)_2-$	48,3	6,6	[28]
22	DF403	H	F	4-CF_3-3-Pyr	CH_3	$-(CH_2)_2-$	18,4	5,0	[28]
23	DF406	H	CH3O	4-CF_3-3-Pyr	CH_3	$-(CH_2)_2-$	10,4	6,9	[28]
24	DF407	F	F	4-CF_3-3-Pyr	CH_3	$-(CH_2)_2-$	54,2	3,9	[28]
25	DF412	H	CH_3	4-CF_3-5-F-Py-3	CH_3	$-(CH_2)_2-$	47,3	3,2	[28]
26	Димебон	H	CH_3	4- CH_3-Py-3	CH_3	$-(CH_2)_2-$	14,4	10,4	[33]
27	EC3030	H	CH_3	Mem	C_2H_5	$-(CH_2)_2-$	65,2	9,2	[29]
28	EC3031	H	H	Mem	CH_3	$-(CH_2)_2-$	19,4	3,1	[16]
29	EC3032	H	F	Mem	CH_3	$-(CH_2)_2-$	57,6	3,9	[16]
30	EC3058	H	CH_3	Mem	C_2H_5	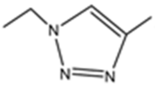	31,3	0,8	[29]
31	EC3061	H	CH_3	Mem	CH_3		60,1	7,8	[29]

No.	Codes	R1	R2	R3	R4	Linker	Activity (inhibition of rate of swelling), %		Ref.
							MEAN	SD	
32	EC3072	H	H	Mem	CH_3		44,7	10,7	[29]
33	EC3148*	H	F	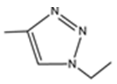	CH_3	-	-2,5	6,3	[30]
34	EC3222	H	F	Ama	CH_3	$-(CH_2)_2-$	42,9	6,7	[16]
35	EC3234	H	CH_3	Ama	CH_3	$-(CH_2)_2-$	48,6	10,5	[16]
36	EC3235	H	H	Ama	CH_3	$-(CH_2)_2-$	40,9	13,3	[16]
37	EC3236	H	CH_3	Mem	CH_3	$-(CH_2)_2-$	52,7	14,1	[16]
38	EC3252	H	CH_3	Ama	CH_3	$-(CH_2)_3-$	50,9	8,4	S1, below
39	EC3253	H	H	Ama	CH_3	$-(CH_2)_3-$	52,5	15,1	S1, below
40	EC3254	H	F	Ama	CH_3	$-(CH_2)_3-$	61,9	9,7	S1, below
41	EC3299	H	Cl	Mem	CH_3	$-CH_2-CH(OH)-CH_2-$	73,5	0,0	[16].
42	СГ1146	H	H	Mem	CH_3	$-(CH_2)_3-$	76,9	3,6	S1, below
43	СГ1147	H	CH_3	Mem	CH_3	$-(CH_2)_3-$	32,6	18,7	[16].
44	СГ1148	H	F	Mem	CH_3	$-(CH_2)_3-$	75,7	4,2	S1, below
45	CA8065	H	H	Mem	CH_3	$-CH_2-C(O)-$	24,0	6,6	[16]
46	CA8759	H	H	Ama	CH_3	$-CH_2-C(O)-$	19,3	4,1	[16]
47	CA8760	H	F	Ama	CH_3	$-CH_2-C(O)-$	29,2	13,5	[16]

No.	Codes	R1	R2	R3	R4	Linker	Activity (inhibition of rate of swelling), %		Ref.
							MEAN	SD	
48	CA8763	H	CH_3	Ama	CH_3	$-CH_2-C(O)-$	16,3	5,0	[16]
49	CA8768	H	F	Mem	CH_3	$-CH_2-C(O)-$	32,0	12,5	[16]
50	CA8769	H	CH_3	Mem	CH_3	$-CH_2-C(O)-$	45,0	17,2	[16]
51	CA8852	H	F	Ama	CH_3	$-CH_2-CH(OH)-CH_2-$	53,3	13,3	[30]
52	CA8867	H	F	Mem	CH_3	$-CH_2-CH(OH)-CH_2-$	69,6	3,2	[30]
53	CA9169	H	CH_3	Mem	CH_3	$-CH_2-CH(OH)-CH_2-$	91,7	4,1	[30]
54	CA9174	H	CH_3	Ama	CH_3	$-CH_2-CH(OH)-CH_2-$	63,6	10,4	[30]
55	CA9175	H	H	Mem	CH_3	$-CH_2-CH(OH)-CH_2-$	70,2	6,3	[30]
56	CA9178	H	CH_3	Ama	C_2H_5	$-CH_2-CH(OH)-CH_2-$	45,5	15,1	[30]
57	CA9179	H	H	Ama	CH_3	$-CH_2-CH(OH)-CH_2-$	35,3	5,9	[30]

* – These compounds were used as bases. All other compounds were used as hydrochlorides.

27. V. B. Sokolov, A. Yu. Aksinenko, T. A. Epishina, T. V. Goreva, E. F. Shevtsova, L. G. Dubova, P. N. Shevtsov, E. V. Rudakova, N. V. Kovaleva, G. F. Makhaeva, S. O. Bachurin, *Russ. Chem. Bull.*, 2018, **67**, 2103; DOI: 10.1007/s11172-018-2335-9

28. Aksinenko, S. Bachurin, S. Sablin, B. Beznosko, V. Sokolov, Fluoro-containing derivatives of hydrogenated pyrido[3,4-b]indoles with neuroprotective and enhancing properties, process for preparing, and use, Pat. US 7935823 B2 (2011).

29. A. Yu. Aksinenko, V. V. Grigoriev, T. V. Goreva, T. A. Epishina, A. V. Gabrelyan, V. P. Fisenko, M. N. Zaharova, and S. O. Bachurin, Binary ligands of glutamate receptors in a series of cyclic indole derivatives. *Russ Chem Bull* **75**, 945–951 (2026). https://doi.org/10.1007/s11172-026-4949-7

30. Aksinenko, A.Y., Goreva, T.V., Epishina, T.A. et al. Molecular design of multitarget neuroprotective agents 6. Synthesis of conjugates of tetrahydro-γ-carbolines and adamantan-1-amines combined via a trimethylene linker. Russ Chem Bull 73, 3677–3683 (2024). https://doi.org/10.1007/s11172-024-4478-1)

31. CAS Registry Number® 19686-05-6 *https://pubchem.ncbi.nlm.nih.gov/compound/209291* (accessed on 20 October 2026).

32. CAS Registry Number® 524-81-2 (Mebhydrolin) *https://pubchem.ncbi.nlm.nih.gov/compound/11150458* (accessed on 20 October 2026)

33. CAS Registry Number® 3613-73-8 (Latrepirdine) *https://pubchem.ncbi.nlm.nih.gov/compound/197033* (accessed on 20 October 2026)

1. General information & materials.

NMR spectra were recorded on Bruker DPX 200 (Germany) (200.13 MHz, ^{1}H) and Quantum-1 spectrometers (China) (399.88 (^{1}H) and 100.54 MHz (^{13}C)) relative to Me_4Si (internal standard, $\delta_H = 0$) in solutions of $CDCl_3$ and DMSO-d_6. Melting points were measured in a glass capillary using a STUART SMP20 apparatus (Cole-Parmer, USA). Elemental composition of the synthesized compounds was determined using a Vario EL cube CHN analyzer (Elementar GmbH) and an AQF-2100H halogen analyzer (Nittoseiko Analytech). Column chromatography was performed on Silica gel 60.0.032—0.063 mm (230—450 mesh, Alfa-Aesar). Copper sulfate and sodium ascorbate were used without prior purification.

2. The synthesis of EC3148

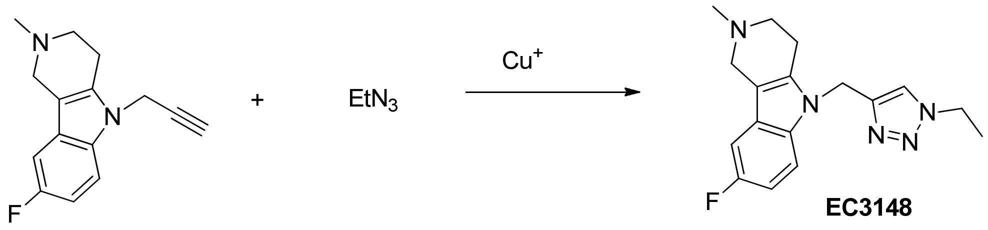

5-((1-ethyl-1*H*-1,2,3-triazol-4-yl)methyl)-8-fluoro-2-methyl-2,3,4,5-tetrahydro-1*H*-pyrido[4,3-b]indole.

The *N*-propynylcarboline (0.1 mmol), $CuSO_4 \bullet 5H_2O$ (0.002 g, 0.01 mmol) in water (0.5 mL), and sodium ascorbate (0.008 g, 0.04 mmol) in water (0.25 mL) were added to a solution of ethylazide (0.1 mmol) in dichloromethane (20 mL) with stirring. The reaction mixture was stirred for 7 h at 40 °C, cooled to room temperature, diluted with dichloromethane (10 mL), and washed with 2% aqueous ammonia (10 mL) and water. The organic layer was separated and dried with Na_2SO_4, The solvent was evaporated *in vacuo*, the residue was subjected to chromatography on silica gel (60 mesh, eluent methanol—chloroform, 1 : 10).

EC3148

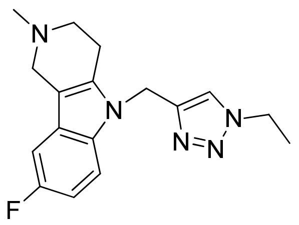

5-((1-Ethyl-1H-1,2,3-triazol-4-yl)methyl)-8-fluoro-2-methyl-2,3,4,5-tetrahydro-1H-pyrido[4,3-b]indole (white solid, 63%). m.p. 98-99 °C. ^{1}H NMR (200 MHz, $CDCl_3$), δ, ppm: 1.45 (t, 3 H, C_2H_5, $^3J_{H,H}$ = 7.3); 2.58 (s, 3 H, NCH_3); 1.29 – 1.51 (m, 4 H, CH_2); 3.65 (s, 2 H, CH_2); 4.27 (quartet, 2 H, C_2H_5, $^3J_{H,H}$ = 7.3); 5.33 (s, 2 H, CH_2); 6.87 (td, 1 H, $C_{Ar}H$, $^3J_{H,F}$ = $^3J_{H,H}$ = 9.1, $^4J_{H,H}$ = 2.4); 7.02 (s, 1 H, CH=CN); 7.05 (dd, 1 H, $C_{Ar}H$, $^3J_{H,H}$ = 9.1, $^4J_{H,H}$ = 2.4); 7.20 (dd, 1 H, $C_{Ar}H$, $^3J_{H,H}$ = 9.0, $^4J_{H,F}$ = 4.4). Calculated (%): C, 65.16; H, 6.43. N, 22.35. $C_{17}H_{20}FN_5$. Found (%): C, 65.32; H, 6.29; N, 22.56.

2.2 General experimental procedures for synthesis of conjugates of γ-carbolines with aminoadamantanes

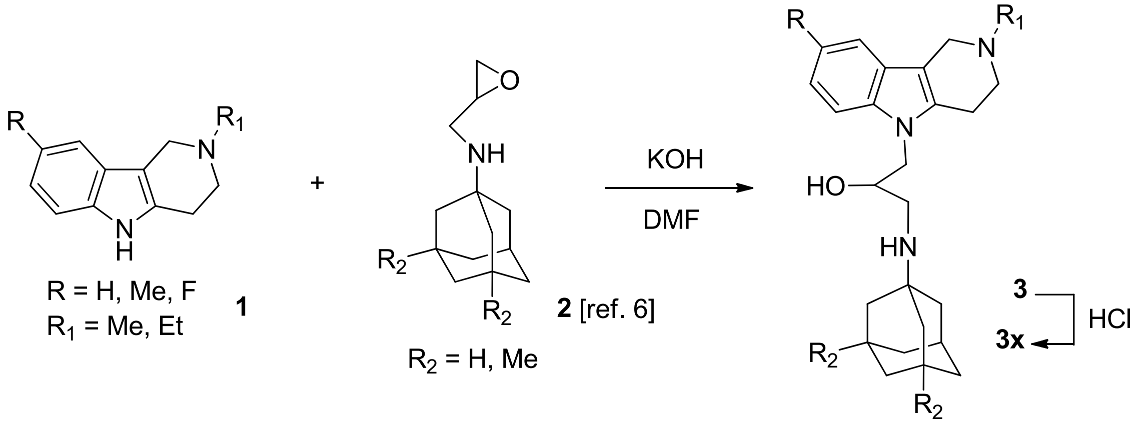

A mixture of carboline **1** (1 mmol), epoxide **2** (1,1 mmol), KOH (1.5 mmol), and DMF (10 mL) was stirred at 90 °C for 4 h. Water was added (50 mL), the mixture was extracted with dichloromethane. The solvent was distilled off, the residue was purified by column chromatography on silica gel (60 mesh, eluent: methanol—chloroform, 1 : 10) yielding **3**.
Aqueous HCl (37%, 0.2 mL) was added to a solution of conjugate **3** (1 mmol) in acetone (1 mL) with stirring, after 1 h the mixture was concentrated, the residue was crystallized from acetone.

Physical and spectral data for compounds

CA9175b

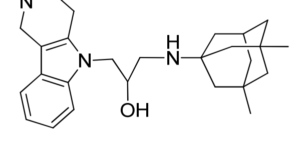

1-((3,5-Dimethyladamantan-1-yl)amino)-3-(2-methyl-3,4-dihydro-1H-pyrido[4,3-b]indol-5(2H)-yl)propan-2-ol (yellow liquid, $CHCl_3$/MeOH = 10:1, 62%). ^{1}H NMR (200 MHz, $CDCl_3$), δ, ppm: 0.80 (s, 6 H, CH_3); 0.90 - 1.15 (m, 6 H, $C_{Ad}H_2$); 1.15 - 1.39 (m, 6 H, $C_{Ad}H_2$); 2.04 (br.s, 1 H, $C_{Ad}H$); 2.31 (s, 3 H,

CH_3N); 2.39 - 2.73 (m, 7 H, , NH + CH_2); 3.48 (d, 1 H, CH_2, $^2J_{H,H}$ = 13.4); 3.62 (d, 1 H, CH_2, $^2J_{H,H}$ = 13.4); 3.78 - 4.05 (m, 4 H, OH + CH + CH_2); 6.95 – 7.17 (m, 2 H, CH_{Ar}); 7.21 – 7.43 (m, 2 H, CH_{Ar}).

CA9175

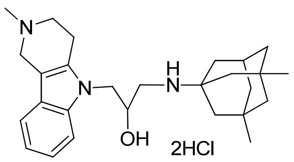

1-((3,5-Dimethyladamantan-1-yl)amino)-3-(2-methyl-3,4-dihydro-1H-pyrido[4,3-b]indol-5(2H)-yl)propan-2-ol dihydrochloride (white solid, 72%). m.p. 253-255 °C. ^{1}H NMR (200 MHz, $CDCl_3$), δ, ppm: 0.88 (s, 6 H, CH_3); 1.04 - 1.22 (m, 2 H, $C_{Ad}H_2$); 1.22 - 1.43 (m, 4 H, $C_{Ad}H_2$); 1.44 - 1.68 (m, 4 H, $C_{Ad}H_2$); 1.69 - 1.78 (m, 2 H, $C_{Ad}H_2$); 2.12 – 2.28 (m, 1 H, $C_{Ad}H$); 2.98 (d, 3 H, NCH_3, $^3J_{H,H}$ = 4.3); 3.11 - 3.38 (m, 2 H, CH_2); 3.08 - 3.35 (m, 3 H, CH_2); 3.39 - 3.62 (m, 1 H, CH_2); 3.68 - 3.88 (m, 1 H, CH_2); 4.00 - 4.44 (m, 3 H, CH_2); 4.62 (d, 1 H, CH_2, $^2J_{H,H}$ = 13.8); 5.86 (br.s, 1 H, OH); 6.98 – 7.30 (m, 2 H, CH_{Ar}); 7.46 (d, 1 H, CH_{Ar}, $^3J_{H,H}$ = 7.3); 7.61 (d, 1 H, CH_{Ar}, $^3J_{H,H}$ = 7.5); 8.82 (quartet, 1 H, NH, $^3J_{H,H}$ = 4.3); 9.12 (br.s, 1 H, NH); 10.99 (br.s, 0.5 H, NH^+); 11.33 (br.s, 0.5 H, NH^+). Calculated (%): C, 65.57; H, 8.36; N, 8.50. $C_{27}H_{41}Cl_2N_3O$. Found (%): C, 65.75; H, 8.12; N, 8.67.

CA9179b

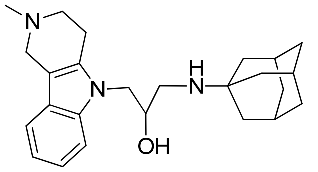

1-((Adamantan-1-ylamino)-3-(2-methyl-3,4-dihydro-1H-pyrido[4,3-b]indol-5(2H)-yl)propan-2-ol (yellow oil, $CHCl_3$/MeOH = 10:1, 46%). ^{1}H NMR (200 MHz, $CDCl_3$), δ, ppm: 1.48 (br.s, 6 H, $C_{Ad}H_2$); 1.51 – 1.75 (m, 6 H, $C_{Ad}H_2$); 2.00 (br.s, 3 H, $C_{Ad}H$); 2.44 (s, 3 H, CH_3N); 2.48 - 2.85 (m, 7 H, NH + CH_2); 3.58 (d, 1 H, CH_2, $^2J_{H,H}$ = 13.3); 3.62 (s, 1 H, OH); 3.69 (d, 1 H, CH_2, $^2J_{H,H}$ = 13.3); 3.82 - 4.05 (m, 3 H, CH + CH_2); 6.98 – 7.20 (m, 2 H, CH_{Ar}); 7.31 (d, 1 H, CH_{Ar}, $^3J_{H,H}$ = 7.9); 7.37 (d, 1 H, CH_{Ar}, $^3J_{H,H}$ = 7.6).

CA9179

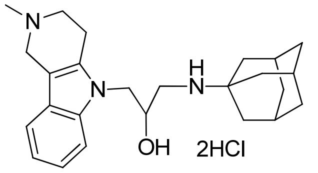

1-((Adamantan-1-ylamino)-3-(2-methyl-3,4-dihydro-1H-pyrido[4,3-b]indol-5(2H)-yl)propan-2-ol dihydrochloride (white solid, 68%). m.p. 239-241 °C. ^{1}H-NMR (200 MHz, DMSO-d_6), δ, ppm: 1.49 – 1.76 (m, 6 H, $C_{Ad}H_2$); 1.77 – 2.03 (m, 6 H, $C_{Ad}H_2$); 2.10 (br.s, 3 H, $C_{Ad}H$); 2.91 (d, 3 H, NCH_3, $^3J_{H,H}$ = 4.3); 3.02 – 3.35 (m, 4 H, CH_2); 3.37 - 3.59 (m, 1 H, CH_2); 3.64 - 3.86 (m, 1 H, CH_2); 4.01 - 4.39 (m, 4 H, CH_2); 4.57 (d, 1 H, CH_2, $^2J_{H,H}$ = 14.5); 5.92 (br.s, 1 H, OH); 6.92 – 7.28 (m, 2 H, CH_{Ar}); 7.48 (d, 1 H, CH_{Ar}, $^3J_{H,H}$ = 7.3); 7.63 (d, 1 H, CH_{Ar}, $^3J_{H,H}$ = 7.5); 8.77 (quartet, 1 H, NH, $^3J_{H,H}$ = 4.3); 9.16 (br.s, 1 H, NH); 11.07 (br.s, 0.5 H, NH^+); 11.32 (br.s, 0.5 H, NH^+). Calculated (%): C, 64.37; H, 7.99; N, 9.01. $C_{25}H_{37}Cl_2FN_3O$. Found (%): C, 64.52; H, 7.73; N, 8.95.

CA9169b

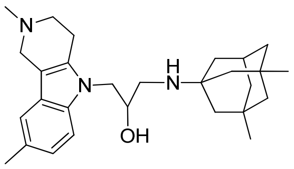

1-(2,8-Dimethyl-3,4-dihydro-1H-pyrido[4,3-b]indol-5(2H)-yl)-3-((-3,5-dimethyladamantan-1-yl)amino)propan-2-ol (yellow oil, $CHCl_3$/MeOH = 10:1, 54%). ^{1}H NMR (200 MHz, $CDCl_3$), δ, ppm: 0.79 (s, 6 H, CH_3); 0.94 - 1.42 (m, 12 H, $C_{Ad}H_2$); 2.04 (br.s, 1 H, $C_{Ad}H$); 2.32 (s, 3 H, CH_3N); 2.41 (s, 3 H, CH_3); 2.35 - 2.83 (m, 7 H, , NH + CH_2); 3.48 (d, 1 H, CH_2, $^2J_{H,H}$ = 13.4); 3.58 (d, 1 H, CH_2, $^2J_{H,H}$ = 13.4); 3.74 - 4.02 (m, 3 H, CH + CH_2); 4.32 (br.s, 1 H, OH); 6.92 (d, 1 H, $C_{Ar}H$, $^3J_{H,H}$ = 8.3); 7.13 (s, 1 H, $C_{Ar}H$); 7.18 (d, 1 H, $C_{Ar}H$, $^3J_{H,H}$ = 8.3).

CA9169

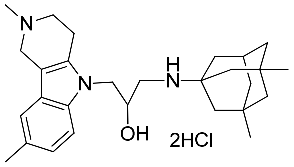

1-(2,8-Dimethyl-3,4-dihydro-1H-pyrido[4,3-b]indol-5(2H)-yl)-3-((3,5-dimethyladamantan-1-yl)amino)propan-2-ol dihydrochloride (white solid, 71%). m.p. 222-224 °C. ^{1}H-NMR (200 MHz, DMSO-d_6), δ, ppm: 0.87 (s, 6 H, CH_3); 1.05 - 1.22 (m, 2 H, $C_{Ad}H_2$); 1.22 - 1.43 (m, 4 H, $C_{Ad}H_2$); 1.44 - 1.68 (m, 4 H, $C_{Ad}H_2$); 1.69 - 1.78 (m, 2 H, $C_{Ad}H_2$); 2.11 – 2.28 (m, 1 H, $C_{Ad}H$); 2.39 (s, 3 H, CCH_3); 2.53 (br.s, 3 H, NCH_3); 2.83 - 3.08 (m, 2 H, CH_2); 3.08 - 3.35 (m, 3 H, CH_2); 3.37 - 3.59 (m, 1 H, CH_2); 3.66 - 3.85 (m, 1 H, CH_2); 4.03 - 4.38 (m, 4 H, OH + CH_2);4.58 (d, 1 H, CH_2, $^2J_{H,H}$ = 14.5); 7.02 (d, 1 H, $C_{Ar}H$, $^3J_{H,H}$ = 8.3); 7.24 (s, 1 H, $C_{Ar}H$); 7.48 (d, 1 H, $C_{Ar}H$, $^3J_{H,H}$ = 8.3); 8.81 (br.s, 1 H, NH); 9.27 (br.s, 1 H, NH); 10.93 (br.s, 0.5 H, NH^+); 11.26 (br.s, 0.5 H, NH^+). Calculated (%): C, 66.13; H, 8.52; N, 8.26. $C_{28}H_{43}Cl_2N_3O$. Found (%): C, 66.85; H, 8.38; N, 8.02.

CA9174b

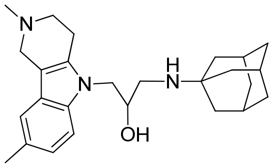

1-(Adamantan-1-ylamino)-3-(2,8-dimethyl-3,4-dihydro-1H-pyrido[4,3-b]indol-5(2H)-yl)propan-2-ol (yellow oil, $CHCl_3$/MeOH = 10:1, 52%). ^{1}H NMR (200 MHz, $CDCl_3$), δ, ppm: 1.42 (br.s, 6 H, $C_{Ad}H_2$); 1.46 – 1.75 (m, 6 H, $C_{Ad}H_2$); 1.99 (br.s, 3 H, $C_{Ad}H$); 2.38 (s, 3 H, CH_3N); 2.41 (s, 3 H, CH_3); 2.42 - 2.84 (m, 7 H, NH + CH_2); 3.50 (d, 1 H, CH_2, $^2J_{H,H}$ = 13.3); 3.62 (d, 1 H, CH_2, $^2J_{H,H}$ = 13.3); 3.83 (br.s, 1 H, OH); 3.85 - 4.03 (m, 3 H, CH + CH_2); 6.92 (d, 1 H, $C_{Ar}H$, $^3J_{H,H}$ = 8.3); 7.15 (s, 1 H, $C_{Ar}H$); 7.18 (d, 1 H, $C_{Ar}H$, $^3J_{H,H}$ = 8.3).

CA9174

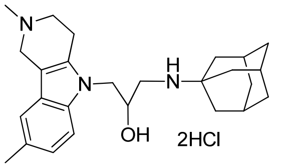

1-(Adamantan-1-ylamino)-3-(2,8-dimethyl-3,4-dihydro-1H-pyrido[4,3-b]indol-5(2H)-yl)propan-2-ol dihydrochloride (white solid, 71%). m.p. 222-224 °C. ^{1}H-NMR (200 MHz, DMSO-d_6), δ, ppm: 1.48 – 1.76 (m, 6 H, $C_{Ad}H_2$); 1.77 – 2.03 (m, 6 H, $C_{Ad}H_2$); 2.13 (br.s, 3 H, $C_{Ad}H$); 2.39 (s, 3 H, CCH_3); 2.98 (d, 3 H, NCH_3, $^3J_{H,H}$ = 4.3); 3.04 – 3.36 (m, 4 H, CH_2); 3.37 - 3.59 (m, 1 H, CH_2); 3.64 - 3.86 (m, 1 H, CH_2); 4.00 - 4.38 (m, 4 H, CH_2); 4.57 (d, 1 H, CH_2, $^2J_{H,H}$ = 14.5); 5.86 (br.s, 1 H, OH); 7.01 (d, 1 H, $C_{Ar}H$, $^3J_{H,H}$ = 8.3); 7.24 (s, 1 H, $C_{Ar}H$); 7.49 (d, 1 H, $C_{Ar}H$, $^3J_{H,H}$ = 8.3); 8.74 (quartet, 1 H, NH, $^3J_{H,H}$ = 4.3); 9.12 (br.s, 1 H, NH); 10.94 (br.s, 0.5 H, NH^+); 11.25 (br.s, 0.5 H, NH^+). Calculated (%): C, 64.99; H, 8.18; Cl, 14.76; N, 8.74. $C_{26}H_{39}Cl_2N_3O$. Found (%): C, 64.85; H, 8.28; N, 8.61.

CA8867b

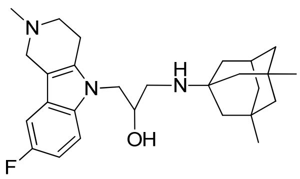

1-((3,5-Dimethyladamantan-1-yl)amino)-3-(8-fluoro-2-methyl-3,4-dihydro-1H-pyrido[4,3-b]indol-5(2H)-yl)propan-2-ol (yellow oil, $CHCl_3$/MeOH = 10:1, 58%). ^{1}H NMR (200 MHz, $CDCl_3$), δ, ppm: 0.79 (s, 6 H, CH_3); 0.94 - 1.42 (m, 12 H, $C_{Ad}H_2$); 2.04 (br.s, 1 H, $C_{Ad}H$); 2.32 (s, 3 H, CH_3N); 2.35 - 2.83 (m, 7 H, , NH + CH_2); 3.48 (d, 1 H, CH_2, $^2J_{H,H}$ = 13.4); 3.58 (d, 1 H, CH_2, $^2J_{H,H}$ = 13.4); 3.74 - 4.02 (m, 3 H, CH + CH_2); 4.32 (br.s, 1 H, OH); 6.84 (td, 1 H, $C_{Ar}H$, $^3J_{H,F}$ = $^3J_{H,H}$ = 9.3, $^4J_{H,H}$ = 2.2); 7.01 (dd, 1 H, $C_{Ar}H$, $^3J_{H,H}$ = 9.6, $^4J_{H,H}$ = 2.2); 7.48 (dd, 1 H, $C_{Ar}H$, $^3J_{H,H}$ = 9.2, $^4J_{H,F}$ = 4.4).

CA8867

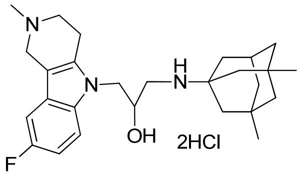

1-((3,5-Dimethyladamantan-1-yl)amino)-3-(8-fluoro-2-methyl-3,4-dihydro-1H-pyrido[4,3-b]indol-5(2H)-yl)propan-2-ol dihydrochloride (white solid, 71%). m.p. 284-285 °C. ^{1}H-NMR (200 MHz, DMSO-d_6), δ, ppm: 0.86 (s, 6 H, CH_3); 1.04 - 1.21 (m, 2 H, $C_{Ad}H_2$); 1.22 - 1.40 (m, 4 H, $C_{Ad}H_2$); 1.44 - 1.67 (m, 4 H, $C_{Ad}H_2$); 1.68 - 1.88 (m, 2 H, $C_{Ad}H_2$); 2.18 (br.s, 1 H, $C_{Ad}H$); 2.93 (d, 3 H, NCH_3, $^3J_{H,H}$ = 4.3); 2.95 - 3.08 (m, 2 H, CH_2); 3.08 - 3.35 (m, 3 H, CH_2); 3.37 - 3.59 (m, 1 H, CH_2); 3.66 - 3.85 (m, 1 H, CH_2); 3.93 - 4.36 (m, 3 H, CH_2);4.53 (d, 1 H, CH_2, $^2J_{H,H}$ = 13.3); 5.87 (br.s, 1 H, OH); 7.01 (t, 1 H, $C_{Ar}H$, $^3J_{H,F}$ = $^3J_{H,H}$ = 9.3); 7.27 (d, 1 H, $C_{Ar}H$, $^3J_{H,H}$ = 9.6); 7.62 (dd, 1 H, $C_{Ar}H$, $^3J_{H,H}$ = 9.2, $^4J_{H,F}$ = 4.4).; 8.85 (quartet, 1 H, NH, $^3J_{H,H}$ = 4.3); 9.31 (br.s, 1 H, NH); 11.06 (br.s, 0.5 H, NH^+); 11.34 (br.s, 0.5 H, NH^+). Calculated (%): C, 63.27; H, 7.87; N, 8.20. $C_{27}H_{40}Cl_2FN_3O$. Found (%): C, 63.42; H, 7.68; N, 8.39.

CA8852b

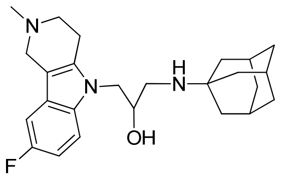

1-((Adamantan-1-ylamino)-3-(8-fluoro-2-methyl-3,4-dihydro-1H-pyrido[4,3-b]indol-5(2H)-yl)propan-2-ol (yellow oil, $CHCl_3$/MeOH = 10:1, 51%). ^{1}H NMR (200 MHz, $CDCl_3$), δ, ppm: 1.42 (br.s, 6 H, $C_{Ad}H_2$); 1.46 – 1.75 (m, 6 H, $C_{Ad}H_2$); 1.99 (br.s, 3 H, $C_{Ad}H$); 2.36 (s, 3 H, CH_3N); 2.42 - 2.84 (m, 7 H, NH + CH_2); 3.46 (d, 1 H, CH_2, $^2J_{H,H}$ = 13.3); 3.58 (d, 1 H, CH_2, $^2J_{H,H}$ = 13.3); 3.81 (br.s, 1 H, OH); 3.85 - 4.08 (m, 3 H, CH + CH_2); 6.84 (td, 1 H, $C_{Ar}H$, $^3J_{H,F}$ = $^3J_{H,H}$ = 9.5, $^4J_{H,H}$ = 2.1); 7.00 (dd, 1 H, $C_{Ar}H$, $^3J_{H,H}$ = 9.5, $^4J_{H,H}$ = 2.1); 7.21 (dd, 1 H, $C_{Ar}H$, $^3J_{H,H}$ = 9.2, $^4J_{H,F}$ = 4.4).

CA8852

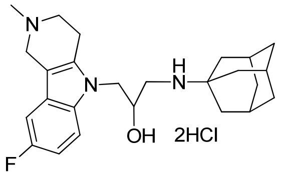

1-((Adamantan-1-ylamino)-3-(8-fluoro-2-methyl-3,4-dihydro-1H-pyrido[4,3-b]indol-5(2H)-yl)propan-2-ol dihydrochloride (white solid, 67%). m.p. 287-289 °C. ^{1}H-NMR (200 MHz, DMSO-d_6), δ, ppm: 1.49 – 1.76 (m, 6 H, $C_{Ad}H_2$); 1.77 – 2.03 (m, 6 H, $C_{Ad}H_2$); 2.10 (br.s, 3 H, $C_{Ad}H$); 2.91 (d, 3 H, NCH_3, $^3J_{H,H}$ = 4.3); 3.02 – 3.35 (m, 4 H, CH_2); 3.37 - 3.59 (m, 1 H, CH_2); 3.64 - 3.86 (m, 1 H, CH_2); 4.01 - 4.39 (m, 4 H, CH_2); 4.57 (d, 1 H, CH_2, $^2J_{H,H}$ = 14.5); 5.92 (br.s, 1 H, OH); 7.01 (t, 1 H, $C_{Ar}H$, $^3J_{H,F}$ = $^3J_{H,H}$ = 9.3); 7.26 (d, 1 H, $C_{Ar}H$, $^3J_{H,H}$ = 9.6); 7.63 (dd, 1 H, $C_{Ar}H$, $^3J_{H,H}$ = 9.2, $^4J_{H,F}$ = 4.4).; 8.79 (quartet, 1 H, NH, $^3J_{H,H}$ = 4.3); 9.21 (br.s, 1 H, NH); 11.07 (br.s, 0.5 H, NH^+); 11.32 (br.s, 0.5 H, NH^+). Calculated (%): C, 61.98; H, 7.49; N, 8.67. $C_{25}H_{36}Cl_2FN_3O$. Found (%): C, 61.82; H, 7.28; N, 8.75.

CA9178b

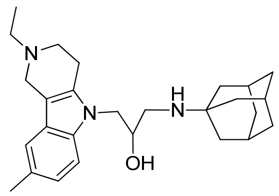

1-(Adamantan-1-ylamino)-3-(2-ethyl-8-methyl-3,4-dihydro-1H-pyrido[4,3-b]indol-5(2H)-yl)propan-2-ol (yellow oil, $CHCl_3$/MeOH = 10:1, 44%). ^{1}H NMR (200 MHz, $CDCl_3$), δ, ppm: 1.25 (t, 3 H, C_2H_5, $^3J_{H,H}$ = 7.2); 1.46 – 1.75 (m, 12 H, $C_{Ad}H_2$); 2.05 (br.s, 3 H, $C_{Ad}H$); 2.46 (s, 3 H, CH_3N); 2.51 - 2.98 (m, 9 H, NH + CH_2); 3.71 (s, 2 H, CH_2); 3.83 (br.s, 1 H, OH); 3.92 - 4.20 (m, 3 H, CH + CH_2); 6.98 (d, 1 H, $C_{Ar}H$, $^3J_{H,H}$ = 8.3); 7.21 (s, 1 H, $C_{Ar}H$); 7.24 (d, 1 H, $C_{Ar}H$, $^3J_{H,H}$ = 8.3).

CA9178

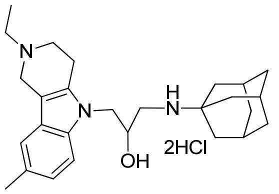

1-(Adamantan-1-ylamino)-3-(2-ethyl-8-methyl-3,4-dihydro-1H-pyrido[4,3-b]indol-5(2H)-yl)propan-2-ol dihydrochloride (white solid, 76%). m.p. 118-120 °C. ^{1}H-NMR (200 MHz, DMSO-d$_6$), δ, ppm: 1.37 (t, 3 H, C_2H_5, $^3J_{H,H}$ = 6.0); 1.51 – 1.76 (m, 6 H, $C_{Ad}H_2$); 1.77 – 2.03 (m, 6 H, $C_{Ad}H_2$); 2.10 (br.s, 3 H, $C_{Ad}H$); 2.38 (s, 3 H, CCH_3); 2.77 – 3.03 (m, 1 H, CH_2); 3.04 – 3.56 (m, 6 H, CH_2); 3.69 - 3.92 (m, 1 H, CH_2); 3.98 - 4.39 (m, 4 H, CH_2); 4.62 (d, 1 H, CH_2, $^2J_{H,H}$ = 14.2); 5.84 (br.s, 1 H, OH); 7.00 (d, 1 H, $C_{Ar}H$, $^3J_{H,H}$ = 8.3); 7.28 (s, 1 H, $C_{Ar}H$); 7.48 (d, 1 H, $C_{Ar}H$, $^3J_{H,H}$ = 8.3); 8.69 (quartet, 1 H, NH, $^3J_{H,H}$ = 4.3); 9.06 (br.s, 1 H, NH); 10.65 (br.s, 0.5 H, NH^+); 11.01 (br.s, 0.5 H, NH^+). Calculated (%): C, 65.57; H, 8.36; N, 8.50. $C_{27}H_{41}Cl_2N_3O$. Found (%): C, 65.65; H, 8.31; N, 8.36.

EC3299b

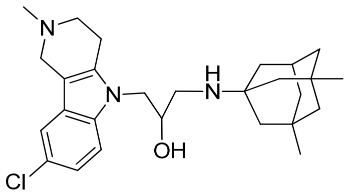

1-(8-Chloro-2-methyl-3,4-dihydro-1H-pyrido[4,3-b]indol-5(2H)-yl)-3-((3,5-dimethyladamantan-1-yl)amino)propan-2-ol (yellow oil, $CHCl_3$/MeOH = 10:1, 65%). ^{1}H NMR (200 MHz, $CDCl_3$), δ, ppm: 0.80 (s, 6 H, CH_3); 0.92 - 1.16 (m, 6 H, $C_{Ad}H_2$); 1.16 - 1.41 (m, 6 H, $C_{Ad}H_2$); 2.06 (br.s, 1 H, $C_{Ad}H$); 2.33 (s, 3 H, CH_3N); 2.41 - 2.82 (m, 7 H, , NH + CH_2); 3.45 (d, 1 H, CH_2, $^2J_{H,H}$ = 13.6); 3.50 (s, 1 H, OH); 3.58 (d, 1 H, CH_2, $^2J_{H,H}$ = 13.6); 3.68 - 4.00 (m, 3 H, CH + CH_2); 7.04 (dd, 1 H, $C_{Ar}H$, $^3J_{H,H}$ = 8.6, $^4J_{H,H}$ = 2.0); 7.2 (d, 1 H, $C_{Ar}H$, $^3J_{H,H}$ = 8.6); 7.31 (d, 1 H, $C_{Ar}H$, $^4J_{H,H}$ = 2.0).

EC3299

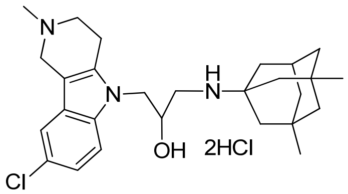

1-(8-Chloro-2-methyl-3,4-dihydro-1H-pyrido[4,3-b]indol-5(2H)-yl)-3-((3,5-dimethyladamantan-1-yl)amino)propan-2-ol dihydrochloride (white solid, 76%). m.p. 271-273 °C. ^{1}H-NMR (200 MHz, DMSO-d$_6$), δ, ppm: 0.87 (s, 6 H, CH_3); 1.04 - 1.22 (m, 2 H, $C_{Ad}H_2$); 1.22 - 1.44 (m, 4 H, $C_{Ad}H_2$); 1.44 - 1.68 (m, 4 H, $C_{Ad}H_2$); 1.68 - 1.92 (m, 2 H, $C_{Ad}H_2$); 2.20 (br.s, 1 H, $C_{Ad}H$); 2.97 (d, 3 H, NCH_3, $^3J_{H,H}$ = 4.3); 3.10 - 3.37 (m, 2 H, CH_2); 3.08 - 3.35 (m, 3 H, CH_2); 3.39 - 3.62 (m, 1 H, CH_2); 3.68 - 3.88 (m, 1 H, CH_2); 3.97 - 4.43 (m, 3 H, CH_2); 4.60 (d, 1 H, CH_2, $^2J_{H,H}$ = 13.8); 5.86 (br.s, 1 H, OH); 7.18 (d, 1 H, $C_{Ar}H$, $^3J_{H,H}$ = 8.6); 7.55(s, 1 H, $C_{Ar}H$); 7.63 (d, 1 H, $C_{Ar}H$, $^3J_{H,H}$ = 8.6); 8.70 (quartet, 1 H, NH, $^3J_{H,H}$ = 4.3); 9.15 (br.s, 1 H, NH); 10.86 (br.s, 0.5 H, NH^+); 11.13 (br.s, 0.5 H, NH^+). Calculated (%): C, 61.30; H, 7.62; N, 7.94. $C_{27}H_{40}Cl_3N_3O$. Found (%): C, 61.45; H, 7.78; N, 7.91.
